# Copolymer Composition and Nanoparticle Configuration Enhance in vitro Drug Release Behavior of Poorly Water-soluble Progesterone for Oral Formulations

**DOI:** 10.2147/IJN.S257353

**Published:** 2020-07-29

**Authors:** Yue Zhang, Rui Zhang, Upulitha Eranka Illangakoon, Anthony Henry Harker, Christopher Thrasivoulou, Maryam Parhizkar, Mohan Edirisinghe, C J Luo

**Affiliations:** 1 Department of Mechanical Engineering, University College London, London WC1E 7JE, UK; 2 UCL School of Pharmacy, University College London, London WC1N 1AX, UK; 3 Department of Physics & Astronomy, University College London, London WC1E 6BT, UK; 4 Cell & Developmental Biology, Division of Biosciences, University College London, London WC1E 6BT, UK

**Keywords:** core-shell nanoparticles, oral formulations, bioavailability, drug delivery, poorly water-soluble drugs, progesterone, poly, lactide-co-glycolide, PLGA, copolymer, coaxial electrospray

## Abstract

**Hypothesis:**

Developing oral formulations to enable effective release of poorly water-soluble drugs like progesterone is a major challenge in pharmaceutics. Coaxial electrospray can generate drug-loaded nanoparticles of strategic compositions and configurations to enhance physiological dissolution and bioavailability of poorly water-soluble drug progesterone.

**Experiments:**

Six formulations comprising nanoparticles encapsulating progesterone in different poly(lactide-co-glycolide) (PLGA) matrix configurations and compositions were fabricated and characterized in terms of morphology, molecular crystallinity, drug encapsulation efficiency and release behavior.

**Findings:**

A protocol of fabrication conditions to achieve 100% drug encapsulation efficiency in nanoparticles was developed. Scanning electron microscopy shows smooth and spherical morphology of 472.1±54.8 to 588.0±92.1 nm in diameter. Multiphoton Airyscan super-resolution confocal microscopy revealed core-shell nanoparticle configuration. Fourier transform infrared spectroscopy confirmed presence of PLGA and progesterone in all formulations. Diffractometry indicated amorphous state of the encapsulated drug. UV-vis spectroscopy showed drug release increased with hydrophilic copolymer glycolide ratio while core-shell formulations with progesterone co-dissolved in PLGA core exhibited enhanced release over five hours at 79.9±1.4% and 70.7±3.5% for LA:GA 50:50 and 75:25 in comparison with pure progesterone without polymer matrix in the core at 67.0±1.7% and 57.5±2.8%, respectively. Computational modeling showed good agreement with the experimental drug release behavior in vitro.

## Introduction

In oral formulations, the aqueous solubility of the drug plays an essential role in drug absorption.^1^ The drug needs to be present in an aqueous solution at a desired concentration^2^ to achieve effective pharmacological response.^3^ However, a large number of drugs have poor water solubility and suffer from low bioavailability and absorption when orally administered.^2^ To achieve the required drug concentration in systemic circulation, oral formulations also need to prevent enzymatic degradation in the gastrointestinal tract to minimize first pass metabolism during which significant loss of drug concentration can occur.^3^

The encapsulation of therapeutics in biodegradable polymer particles is one of many strategies to enable sustained and controllable drug release while preserving nonreleased drugs from rapid biodegradation.^4^ It has been demonstrated that biodegradable polymers used to produce drug-loaded microparticles result in the drug being molecularly dispersed in amorphous form, thereby significantly improving the bioavailability of non-water-soluble drugs.^5^ Over the past two decades, researchers have been exploring biodegradable nanomaterials for drug delivery^6^–^15^ in which nanoparticles are frequently employed as oral, injectable or inhalable drug delivery systems whereby the drug is continuously released from the particles over a defined period of time. Drug-loaded nanoparticles fabricated with biodegradable polymers are promising candidates for oral delivery of poorly water-soluble drugs. In biodegradable nanoparticle systems, drugs are shielded from enzymatic degradation of the gastrointestinal tract, thereby minimizing the undesirable first pass metabolism during which significant loss of drug concentration can occur before reaching systemic circulation.^7^ Moreover, by incorporating poorly water-soluble drugs into a polymeric matrix, amorphous state can be achieved to enhance drug dissolution rate,^16^ making this a practical oral delivery strategy.

Progesterone, a poorly water-soluble hormone (16.8 μg per mL in distilled water, 15.1 μg per mL in 0.9% aqueous saline at room temperature),^17^ is tested in this work as the model drug. Progesterone is an endogenous steroidal hormone essential in reproduction and mainly prescribed for menopausal symptoms as part of hormonal replacement therapies.^18^ In addition, progesterone is used to prevent preterm birth, as it is involved in building uterine quiescence and essential for maintaining pregnancy.^19^ Preterm birth, defined by the World Health Organization as birth before 37 weeks of pregnancy,^20^,^21^ is the leading cause of neonatal morbidity and mortality in infants.^21^ Progesterone can be administered orally, parentally (intramuscularly and subcutaneously) and/or topically as a cream of vaginal gel, of which the preferred route for administration is oral delivery. However, oral delivery of pure progesterone suffers from high first pass metabolism, compromising the efficacy of the therapy; the bioavailability of progesterone is also limited owing to short half-life and low water solubility.^22^ Conversely, intramuscular/subcutaneous injections may bypass first pass metabolism but cannot achieve good patient compliance due to the need for frequent daily injections which can be painful and challenging to comply.

Drug carriers such as polymer particles loaded with progesterone can be administered via depot injection in which the therapeutic is deposited in a localized mass via intramuscular, subcutaneous or intradermal injection. The method allows for continuous drug release for a prolonged period of time in a sustained manner using a biodegradable carrier.^23^ It has the advantage of improving patient compliance thanks to the reduced need for repeat treatment.

Among the different materials available for producing solid polymer particle drug carriers, copolymers are often good excipients as controlled drug delivery vehicles since their physicochemical properties can be readily varied by altering their molecular weight, functional chemical groups and monomer ratios, thereby achieving desirable drug release behavior.^6^,^24^ For this purpose, poly(lactide-co-glycolide) (PLGA) copolymer is chosen as the model polymer drug carrier in this study. PLGA has been widely employed as a drug carrier in pharmaceutical applications because of its tailorable biodegradability, biocompatibility and physicochemical adjustability, FDA approval and ready commercial availability over a good range of molecular weights and copolymer compositions.^25^

Polymeric nanoparticles can be produced via different methods including emulsification,^26^ solvent evaporation,^27^ spray-drying,^28^ microfluidics^29^ and electrospray.^30^ Each method has advantages and drawbacks. For example, while emulsification has the feasibility for large-scale production, it suffers from broad particle diameter distribution and low encapsulation efficiency— the encapsulation of hydrophobic and hydrophilic drugs in a single vehicle usually involves multiple emulsion steps, resulting in the drug waste and sometimes even drug inactivation.^27^ While spray-drying enables pure drug nanonization and reproducible production of drug carriers, its high temperature and fast drying rates limit the encapsulation of temperature-sensitive drugs.^31^ Furthermore, microfluidics offers production of monodispersed particles with coefficient of variation below 2%, but the technique is generally low yield and highly prone to device blockages in the capillary channels used to generate nanoparticles. In solvent evaporation approaches non-degradable surfactants can integrate with the drug which can induce unwanted effects.^32^,^33^ In comparison, coaxial electrospray has its unique advantages. For instance, it can encapsulate both hydrophilic and hydrophobic constituents in a single step with high encapsulation efficiency, it can be scaled up through well established multi-needle equipment and has good reproducibility and relatively precise control over nanoparticle diameter distribution. Moreover, drug-blended particles often have issues of initial burst release, and coaxial electrospray is a useful technique to load drugs in core-shell or multilayered configurations to enhance the sustainability of the drug delivery system.^34^,^35^

During electrospray, a liquid with sufficient electrical conductivity is pumped into a capillary and charged by a high voltage supply to generate a sufficiently high potential with respect to a ground electrode, and forms a spray of monodisperse droplets at the tip of the capillary.^36^ The solvent evaporates during the flight of the droplets toward a grounded collector, resulting in near-monodisperse particles in a single-step process. Because of Coulombic repulsion, particle aggregation can be avoided due to self-dispersal.^37^

The objective of this study is to systematically investigate the effects of PLGA polymer composition and electrosprayed nanoparticle configurations on the resulting progesterone drug release behavior in vitro. Six formulations of monodisperse nanoparticles of varying particle configurations (eg mono or core-shell, pure drug encapsulation or encapsulating drug dispersed in polymer matrix) and PLGA copolymer compositions in term of lactide:glycolide (LA:GA) ratio (75:25 or 50:50) were fabricated and tested to determine their influence on drug release behavior. Specifically, two monolayer types of PLGA 75:25 or 50:50 with co-dissolved progesterone in the polymer matrix (1-layer-75 and 1-layer-50), two core-shell types containing pure progesterone core with no PLGA in the core (2-layer^1^-50 and 2-layer^1^-75) and two core-shell types containing progesterone co-dissolved with PLGA matrix in the core (2-layer^2^-50 and 2-layer^2^-75) were characterized. We studied the nanoparticles in terms of morphology, molecular crystallinity, encapsulation efficiency and in vitro drug release and modeled the drug release behavior computationally which achieved good agreement with the experimental data.

## Materials and Methods

### Materials

Progesterone, compressed PBS tablet, gold nanoparticles (15 nm diameter, Optical Density 1, stabilized suspension in 0.1 mM PBS), acetone and N, N-dimethylacetamide (DMAc), Evans blue, and Nile red were supplied by Sigma-Aldrich Co. (Poole, UK) at the highest available purity and used as received. PLGA 50:50 (Mw 17,000 g mol^−1^) and 75:25 (Mw 17,000 g mol^−1^) were purchased from Corbion (Amsterdam, The Netherlands) and used as received.

### Particle Fabrication

**Figure 1:**
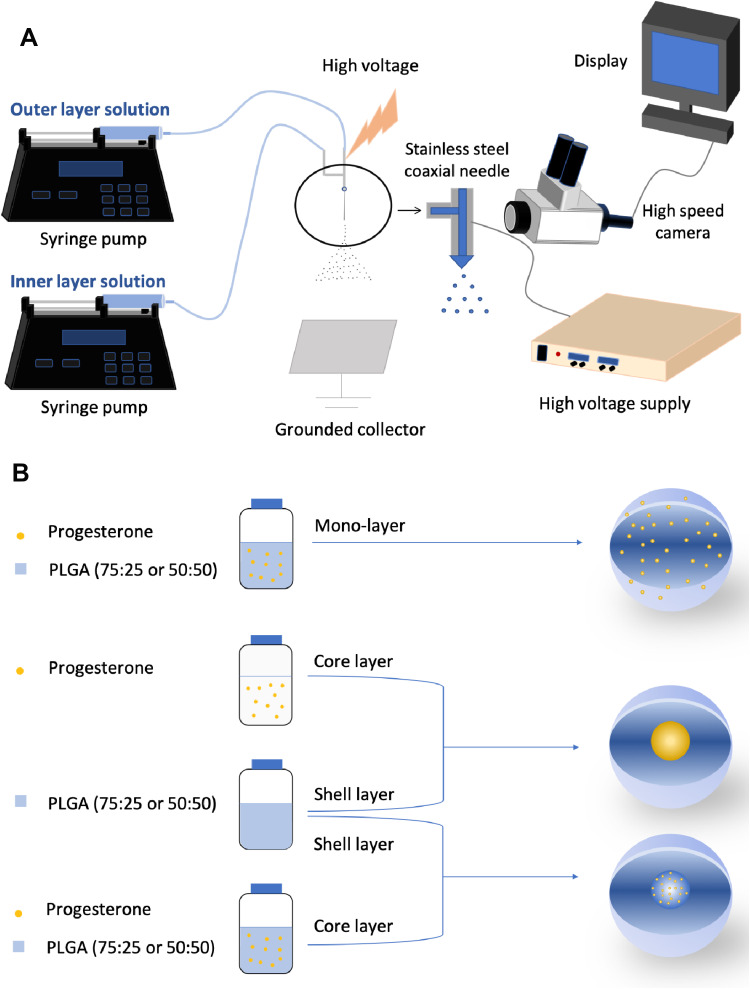
Schematic illustration of (**A**) coaxial electrospray setup; (**B**) formulations of progesterone-loaded nanoparticles.

Progesterone-loaded PLGA nanoparticles were synthesized using different electrospray systems (Figure 1). Acetone and DMAc were mixed in a volume ratio of 7:3. Polymer was dissolved in acetone/DMAc mixture, stirred for 30 min to make PLGA solutions for all formulations (Table 1). Progesterone in acetone/DMAc mixture was used as the inner solution for formulation 2-layer^1^-50 and 2-layer^1^-75 and was added to polymer solution to make the inner solution for formulation 2-layer^2^-50 and 2-layer^2^-75 and single solution for formulation 1-layer-50 and 1-layer-75. For all formulations, the ratio of polymer: drug (w:w) was always kept constant at 40:1. The coaxial nozzle assembly comprises two stainless-steel needle capillaries with inner and outer diameters of 0.254 and 0.457 mm for the inner capillary, and 0.84 and 1.24 mm for the outer capillary, respectively. Single needle electrospray with internal and external needle diameter of 0.84 and 1.24 mm, respectively, was used to fabricate monolayered nanoparticles.

**Table 1:** Physicochemical Characteristics of the Solutions Used for Particle Formation. In the Case of Viscosity, Electrical Conductivity and Density the Error was Negligible

Formulations	Particle Configurations	Surface Tension (mN m^−1^)	Viscosity (mPa s)	Electrical Conductivity (×10^−5^ S m^−1^)	Density (kg L^−1^)
2-layer^1^-50	Shell: 40 gL^−1^ PLGA (50:50) Core: 1 gL^−1^ progesterone	Shell: 30.4±0.3 Core: 27.1±0.2	Shell: 0.7 Core: 0.6	Shell: 4 Core: 3	Shell: 8.7 Core: 8.6
2-layer^2^-50	Shell: 20 gL^−1^ PLGA (50:50) Core: 20 gL^−1^ PLGA (50:50) + 1 gL^−1^ progesterone	Shell: 28.8±0.2 Core: 27.8±0.4	Shell: 0.6 Core: 0.6	Shell: 3 Core: 3	Shell: 8.6 Core: 8.6
1-layer-50	Single: 40 gL^−1^ PLGA (50:50) + 1 gL^−1^ progesterone	30.2±0.3	0.6	4	8.7
2-layer^1^-75	Shell: 40 gL^−1^ PLGA (75:25) Core: 1gL^−1^ progesterone	Shell: 29.8±0.4 Core: 27.1±0.2	Shell: 0.8 Core: 0.6	Shell: 4 Core: 5	Shell: 8.7 Core: 8.6
2-layer^2^-75	Shell: 20 gL^−1^ PLGA (75:25) Core: 20 gL^−1^ PLGA (75:25) + 1 gL^−1^ progesterone	Shell: 28.9±0.5 Core: 28.1±0.3	Shell: 0.5 Core: 0.5	Shell: 3 Core: 3	Shell: 8.6 Core: 8.6
1-layer-75	Single: 40 gL^−1^ PLGA (75:25) + 1 gL^−1^ progesterone	31±0.6	0.7	4	8.5

The corresponding liquids were loaded into a 10 m plastic syringe and fed into thecapillaries at constant flow rate of 0.18 mL h^−1^ by independently controlled syringe pumps (Harvard PHD 4400; Harvard Apparatus, Holliston, MA, USA). A high voltage power supply (Glassman Europe Ltd, Tadley, Hampshire, UK) was used to generate a high positive electrical potential between the nozzle tip and grounded collector varying from 12.0 to 14.0 kV. A Leica DMS300 camera (Leica Microsystems, Wetzlar, Germany) was used to monitor the cone-jet formed during particle fabrication. The particles were collected, after stable cone-jet mode jetting, onto a sheet of grounded collector kept 200 mm below the needle exit. All experiments were conducted at an ambient temperature (20°C) and relative humidity of 40–55%.

### Characterization of Solutions

The density (*ρ*) of all the solutions were measured using a 25 mL standard density bottle (DIN ISO 3507-Gray-Lussac). A Kruss tensiometer (Tensiometer K9, Hamburg, Germany) was used to measure the surface tensions (γ). A U-tube viscometer (VWR, size E) was used to measure the viscosity (μ). A conductivity probe (Jenway 3540 pH/conductivity meter; Fisher Scientific, Loughborough, Leicestershire, UK) was used to estimate the electrical conductivity (κ). The characterization of all solutions was conducted at room temperature and the values are summarized in Table 1.

### Microscopy

Particles were first collected on microscopic glass slides for optical microscopy (Nikon Eclipse ME 600; Nikon Instruments, Melville, NY, USA). They were then collected on foil and further analyzed using a field emission scanning electron microscope (SEM; Hitachi and FEI Helios Nanolab G3 660 UC; Hitachi Ltd, Tokyo, Japan) for particle diameter and morphology analysis. A super-resolution confocal microscope (Zeiss LSM 980 Multiphoton Airyscan 2; Carl Zeiss Ltd, Cambridge, UK) was used in oil-immersion mode with a 63X objective lens to observe the presence of the core-shell layers in the particle structure. To distinguish the two layers, the inner layer structure encapsulated Nile red dye (excited at 690 nm) and the outer layer encapsulated Evans blue dye (excited at 480 nm). Z-stacks were acquired at 0.1 µm intervals and orthogonal projections were generated to visualize the signals of the two layers. Particles were randomly chosen to measure the diameter using ImageJ, a public domain Java image-processing program.

### Production Yield and Drug Encapsulation Efficiency

The production yield of the processing technique was calculated as the ratio of the mass of dried particles collected (M_c_) to the theoretical mass of particles sprayed during the collection time (M_i_).

(1) $${\rm{production \ yield }}\left({\rm{\% }} \right) = \left({{{{{\rm{M}}_{\rm{c}}}} \over {{{\rm{M}}_{\rm{i}}}}}} \right) \times 100{\rm{\% }}$$

(2) $${{\rm{M}}_{\rm{i}}} = {\rm{polymer concentration }} \times {\rm{ flow rate }} \times {\rm{ collection time}}$$

The drug loading in the composite particles produced by single and coaxial electrospray was evaluated by measuring the amount of drug in the collected particles. Particles loaded with 10 mg progesterone was dissolved in DMAc (10 mL) followed by addition of DMAc and PBS mixture (1:9 v/v). The drug concentration was measured by analyzing its absorbance (224 nm) in a UV-visible spectrophotometer.

(3) $${\rm{Encapsulation\ Efficiency}} = {\matrix{ {\rm{mass}}\,{\rm{of}}\,{\rm{actual}}\,{\rm{drug}}\,{\rm{loaded}}\,\, \hfill \cr \,\,\,\,\,\,\,\,\,\,\,\,\,\,{\rm{in}}\,{\rm{particles}} \hfill \cr} \over \matrix{ {\rm{mass}}\,{\rm{of}}\,{\rm{drug}}\,{\rm{used}}\,{\rm{in}}\,\, \hfill \cr {\rm{particle}}\,{\rm{fabrication}} \hfill \cr} } \times 100\rm \% $$

### X-ray Powder Diffraction

X-ray powder diffraction (XRD) patterns of samples were obtained on a Miniflex 600 diffractometer (RigaKu, Tokyo, Japan). The X-rays were generated by a cathode ray tube filtered to produce monochromatic radiation directed towards the sample. Cu Kα radiation was used with wavelength 1.5406Å and graphite monochromatic filtering were at a tube voltage of 40 mV and tube current of 15 mA. The interaction of the incident rays with the sample produces constructive interference (and diffracted rays). The diffracted intensity was recorded in the 2θ angle range from 3 to 40° at a scanning speed of 0.02° min^−1^.

### Fourier Transform Infrared Spectroscopy

Attenuated total reflectance Fourier transform infrared (FTIR) analysis was carried out on a Spectrum 100 FTIR spectrometer (PerkinElmer, Waltham, MA, USA). The resolution was set at 1 cm^−1^ with a scanning range of 650–4000 cm^−1^.

### In vitro Drug Release of Progesterone

To investigate the release profile of progesterone-loaded nanoparticles, in vitro progesterone dissolution study was carried out in PBS (pH 7.4) for five days. Fresh PBS solution was made by dissolving one compressed PBS pellet in 200 mL of deionized water. Forty-five milligrams of dried drug-loaded particles from each sample was placed in a dissolvable polymer capsule. To investigate how the PLGA matrix affects the solubility of poorly water-soluble progesterone after electrospray, an equivalent amount of progesterone was placed into a dissolvable polymer capsule and studied as a control group under the same release conditions. The capsule was then put in a stainless-steel sinker basket. The sinker basket was submerged into a 50 mL PBS container, and subsequently incubated in a water bath (37°C, 120 rpm). At selected time points, samples (3 mL) were collected from each solution exchanged with an equal volume of fresh PBS. The samples were filtered (membrane pore diameter of 0.22 µm) to avoid any impurities and interference in absorbance. Three replicates of each sample were analyzed for drug concentration and measured using a UV-visible spectrophotometer at 224 nm. A calibration equation was developed to calculate the progesterone of unknown samples by measuring the absorbances of known concentrations of progesterone (5, 10, 15 and 20 µg mL^−1^).

## Results and Discussion

### Fabrication of Nanoparticles

Different formulations of progesterone-loaded nanoparticles (Table 1) were fabricated using single and coaxial needle electrospray under the same operating conditions, to produce nanoparticles with similar diameters (Table 2). Applied voltage was optimized to achieve a stable cone-jet to enable particle production of uniform diameter distribution. The effects of PLGA copolymer ratio and particle configuration were characterized and in vitro drug-release behavior was experimentally and computationally analyzed.

**Table 2:** Operating Conditions and Corresponding Particle Characterization

Solution Formulations	Processing Parameters		Average Particle Diameter (nm)	Yield (%)	Encapsulation Efficiency (%)
	Flow Rate (mL h^−1^)	Voltage (kV)			
2-layer^1^-50	Core: 0.18	12.0	588.0±92.1	85±2	95±3
	Shell: 0.18				
2-layer^2^-50	Core: 0.18	13.1	472.1±54.8	86±3	94±2
	Shell: 0.18				
1-layer-50	Single: 0.18	12.8	489.1±67.8	88±4	94±3
2-layer^1^-75	Core: 0.18	12.5	483.2±62.5	84±2	93±3
	Shell: 0.18				
2-layer^2^-75	Core: 0.18	13.7	480.1±84.7	85±3	95±2
	Shell: 0.18				
1-layer-75	Single: 0.18	12.2	535.9±91.6	87±2	92±3

### Particle Diameter and Geometry

**Figure 2:**
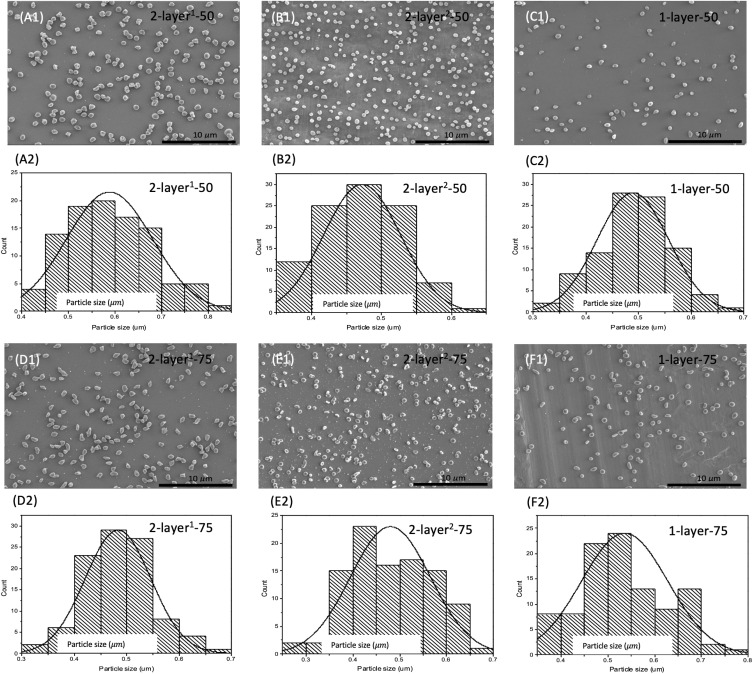
SEM images and corresponding diameter distribution of drug-loaded nanoparticles produced using coaxial needle electrospray (**A**) 2-layer^1^-50, (**B**) 2-layer^2^-50, (**D**) 2-layer^1^-75, (**E**) 2-layer^2^-75 and single needle electrospray (**C**) 1-layer-50 and (**F**) 1-layer-75, respectively. Scale bars: 10 µm.

Single-layered and double-layered nanoparticles were produced using PLGA with copolymer ratios of 50:50 and 75:25 to investigate the effect of the copolymer ratio and the layered core-shell formulation on the resultant particle morphology and release behavior. A constant flow rate of 0.18 mL was used for both mono- and double-layered nanoparticles and the voltage required to form a stable cone jet was between 12.0 kV and 14.0 kV. The particle diameter and morphology were studied using SEM, and the representative SEM images of each formulation are presented in Figure 2. All particles produced showed smooth surface morphology. It was observed that at a constant flow rate, the variation of the particle composition results in the requirement of a small variation in the voltage needed to achieve cone jet. While the variation is between 12 and 14 kV, smaller particles (108 nm smaller) were produced with higher applied voltage, the difference is statistically significant (*p*=1.5x10^−15^) while the outer surface morphology of the particles remained unchanged. Smaller particles were produced with higher applied voltage while the outer surface of the particles stayed smooth. The results showed that the copolymer ratio and capillary configuration have no considerable influence on particle diameter.

**Figure 3:**
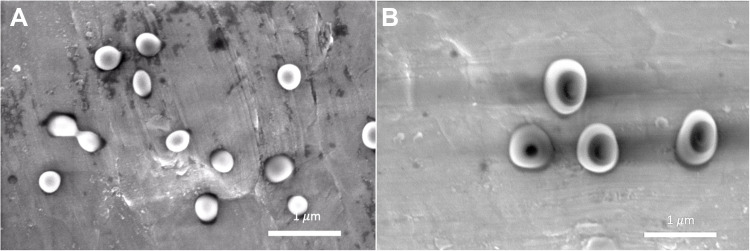
SEM images of gold labelled, drug-loaded nanoparticles. (**A**) monolayer nanoparticles and (**B**) double-layered nanoparticles. Scale bar: 1 µm.

SEM images (Figure 2) showed that the solution from PLGA copolymer ratio of 50:50 for single and coaxial electrospray resulted in the formation of spherical particles, while solutions from copolymer ratio of 75:25 produced elongated or irregular particles. These results were further proved in an earlier study.^30^ To investigate the effect of copolymer formulation and particle configuration on progesterone release profile, particles obtained from all formulations are required to have similar diameters (472.1±54.8 to 588.0±92.1 nm). The drug-loaded nanoparticles were observed by SEM (Figure 3) in which the outer PLGA layer was labelled using gold nanoparticles.

**Figure 4:**
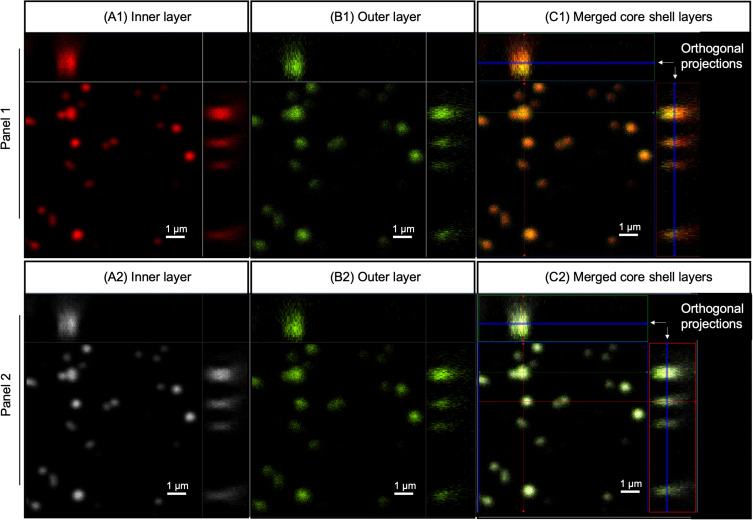
Confocal images of core shell nanoparticles, where the core (**A**) and shell layers (**B**) were labeled in the nanoparticles. Panel 1 shows the inner (**A1**) and outer layer (**B1**) in red and green, respectively. In the merged image (**C1**), the green outer layer overlapped with the red core color resulting in a yellow color. Therefore, panel 2 shows the inner (**A2**) and outer layer (**B2**) in white and green, respectively, to allow better distinction of the two layers in the merged image (**C2**). Scale bar: 1 µm.

To confirm the presence of the two core-shell layers, a multiphoton super-resolution confocal microscope equipped with Airyscan 2 (Zeiss LSM 980) was used to observe the nanoparticles with the inner layer encapsulating Nile red dye, excited at 690 nm so as not to overlap with the Evans blue peak; and the outer layer encapsulating Evans blue dye, excited at 480 nm. Lamda laser scan confirmed the presence of two distinct excitation peaks corresponding to the two dyes in the core and shell layers. Figure 4 shows the confocal images of core-shell nanoparticles, where the core (Figure 4A) and shell layer (Figure 4B) were presented in red and green, respectively. The merged core shell layers with orthogonal projection confirmed the presence of two distinct layers in the nanoparticles (Figure 4C). In the merged image (Figure 4 C1), the green outer layer bled into the red core color and resulting in a yellow colored image. Therefore, Figure 4 panel 2 presented the inner (A2) and outer layer (B2) in white and green, respectively, to allow better distinction of the two layers in the merged image (C2).

### X-Ray Diffraction

**Figure 5:**
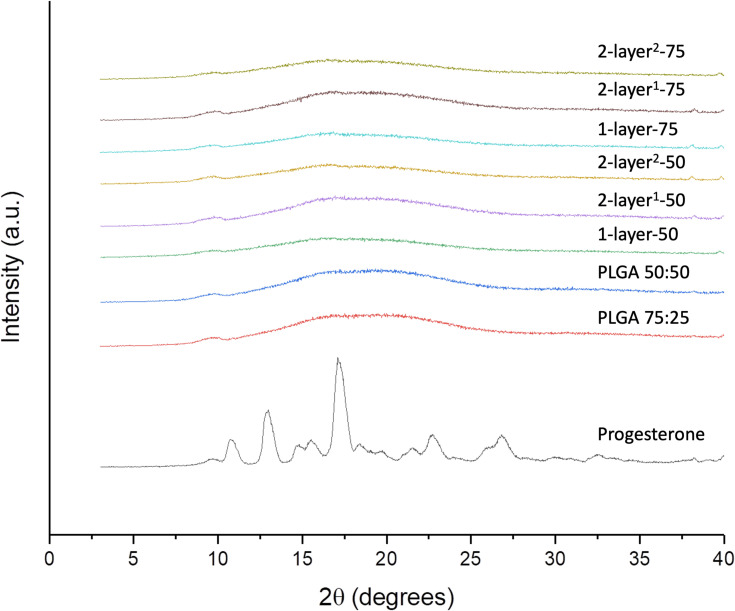
XRD patterns of PLGA (50:50), PLGA (75:25), progesterone, progesterone-loaded particle formulations 1-layer-50, 2-layer^1^-50, 2-layer^2^-50, 1-layer-75, 2-layer^1^-75 and 2-layer^2^-75.

The physical form of the drug, polymer and drug-loaded nanoparticles were tested using XRD and the patterns are shown in Figure 5. The diffractogram of progesterone shows strong characteristic peaks at 2θ values of 10.48, 12.64 and 16.9 degrees, which indicate crystallinity in pure form.^30^ Both PLGA 75:25 and 50:50 diffractograms have the characteristics of amorphous material. Nanoparticles in all formulations showed no crystalline peaks, indicating complete amorphization of progesterone. In comparison with patterns from the pure polymers without drug, the drug-loaded particles all exhibit a weak elevation in intensity at 2θ value of 16.9 degree, which correspond to the strongest peak exhibited by pure progesterone, indicating molecular interaction and dispersion of the drug in the polymer matrix.

### Fourier Transform Infrared Spectroscopy

**Figure 6:**
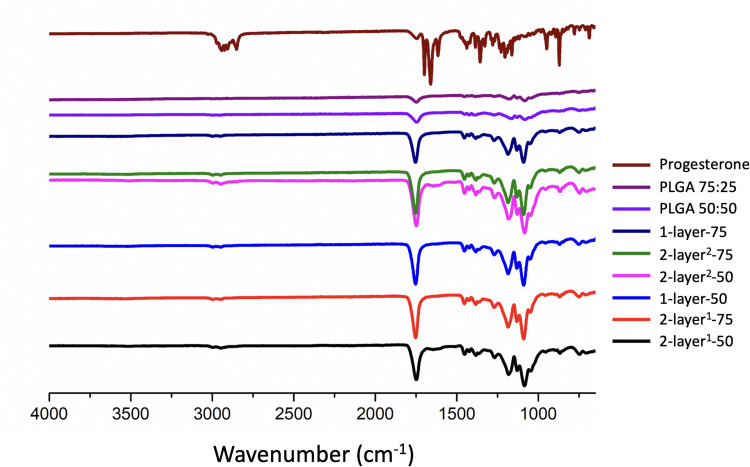
FTIR spectra of progesterone, PLGAs with different copolymer ratios (75:25 and 50:50) and electrosprayed progesterone-loaded particles.

FTIR spectra of pure progesterone and progesterone-loaded particles are shown in Figure 6. The pure progesterone sample showed characteristic peaks at 3000 cm^−1^ for the hydrogen band. The peaks at 1665 and 1697 cm^−1^ are respectively assigned to carbonyl-stretching bands of vinyl keto group in C_3_ and aliphatic methyl keto group in C_20_ in progesterone.^30^ The changes in the carbonyl-stretching bands are important for the interpretation of the molecular state of the drug. PLGA polymers showed carbonyl-stretching at 1750 cm^−1^, and the bands at this position shifted for all drug-loaded particles, indicating drug-PLGA molecular interaction. In the spectra of the nanoparticles, the stretching bands of the carbonyl group are reduced. These spectral changes indicate the formation of the intermolecular hydrogen bonds between progesterone and the PLGA polymer chain.

### In vitro Drug Release Study

**Figure 7:**
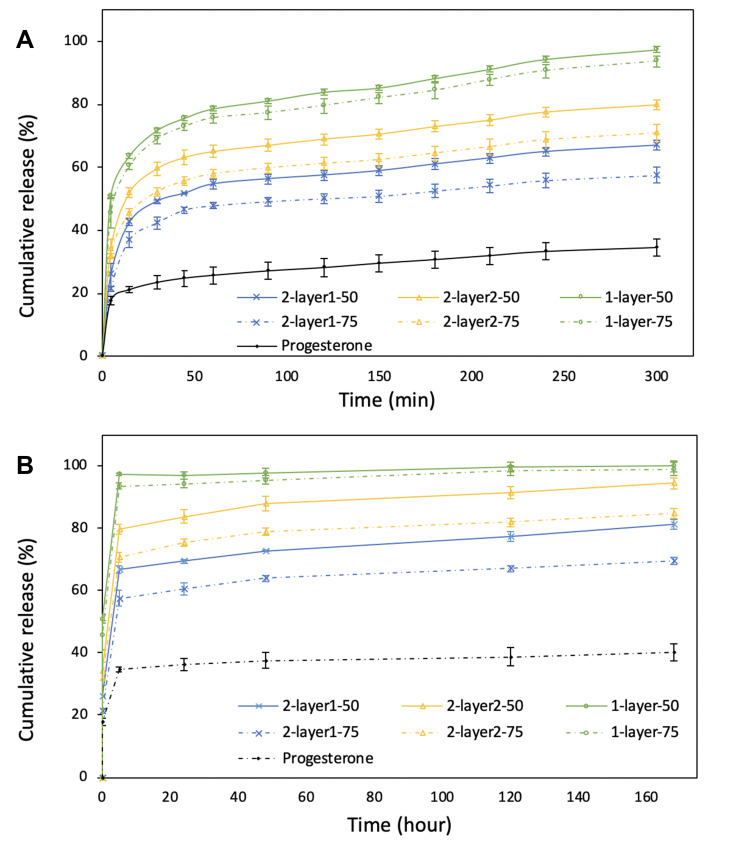
Release profile of progesterone from various formulations in comparison with progesterone dissolution on its own. (**A**) The release profile of progesterone from first five hours. (**B**) The release profile over seven days.

The cumulative drug release behavior of progesterone from all formulations in this work showed a biphasic pattern where phase I is described as burst release, and phase II sustained release (Figure 7). The burst release phase I is attributed to three reasons based on the literature:^38^ firstly due to drug molecules embedded on the particle surface; secondly due to drug released from the pores near and beneath the particle surface; and thirdly due to diffusion of drug in the inner side of the particle through interconnected pores as water infiltrates and fills the particle pores throughout the polymeric matrix. For small hydrophobic molecules such as progesterone with a molecular weight of 314.46 g mol^−1^, the molecules can transport through the polymer matrix of the nanoparticles during drying. The sustained release phase II in this study is attributed to drug diffusion, either through water-filled pores or water-swelled components of the polymer matrix.^39^ For this water diffusion-controlled drug release, varying the rate of water uptake and swelling ability of the polymer chain through increase in hydrophilicity of the copolymer composition can increase the molecular diffusion rate within the polymer matrix, thereby influencing the drug release behavior.^40^

Hence, variations in the hydrophilicity of the PLGA copolymer composition by changing the presence of the hydrophobic lactide monomer weres found to be one of the key factors affecting the drug release behavior in the nanoparticles. Specifically, particles from 1-layer and 2-layer electrospray processes were PLGA-based composites with the same drug to polymer ratio and similar particle diameters, yet they exhibited significant differences in drug release behavior when the PLGA copolymer ratio was varied. In a previous study, we proposed a drug delivery system comprising microparticles uniformly loaded with progesterone in PLGA polymer matrix and controlled the drug release rate through compositional variations in PLGA monomer ratios and concentrations.^30^ The results in this work demonstrate that the PLGA monomer ratio effect on drug release also applies to nano-diameter particles. For both single and double-layered PLGA particles, the more hydrophobic polymer with higher LA:GA ratio (PLGA 75:25) resulted in slower cumulative drug release which is attributed to the decrease in hydrophilicity of PLGA 75:25 in comparison with PLGA 50:50 (Figure 7). Water-uptake and polymer hydration occurred immediately for PLGA 50:50 particles upon immersion in water or upon administration in vivo.^41^ PLGA with a higher GA copolymer ratio is considered to be more hydrophilic and thus has a higher water absorption rate^30^ which causes pore formation and results in an increase in drug diffusion.^42^

Furthermore, the quantities of drug released in the first 30 min from monolayer nanoparticles 1-layer-50 and 1-layer-75 were 71.7±1.3% and 68.8±1.2%, respectively. In contrast, progesterone release from the core-shell nanoparticles displayed lower initial burst release rate in the first 30 min (Figure 7A, 49.2±0.6%, 59.6±2.2%, 42.4±2.0% and 52.1±1.3% for 2-layer^1^-50, 2-layer^2^-50, 2-layer^1^-75 and 2-layer^2^-75, respectively) and retained more drug in the polymer matrix at two days (Figure 7B, cumulative release of 72.8±2.9%, 88.0±1.3%, 63.9±2.7% and 78.9±3.9%, for 2-layer^1^-50, 2-layer^2^-50, 2-layer^1^-75 and 2-layer^2^-75, respectively). The decreased initial drug release of the core shell structures in comparison with the monolayer particles is attributed to the different configuration of the particles. Because of the presence of a polymer shell, the core shell particles encapsulate progesterone in the core with a hard shell of PLGA, resulting in a region of lower drug density on the particle surface. Here, we further note that the progesterone release behavior is different between the two core shell configurations aside from copolymer ratio. The drug release rate is lower in formulation 2-layer^1^-50 and 2-layer^1^-75 in which only the outer layer contains the PLGA matrix, in comparison with those of 2-layer^2^-50 and 2-layer^2^-75 where both the core and shell layers contain PLGA (*p*-value=0.0015). The formulations with decreased initial release of progesterone may be beneficial for developing nanomedicine strategies to enable sustained drug release in novel oral formulations using biomaterials.

In addition, the control group using pure progesterone without PLGA released 40.9% drug after seven days, which is significantly less than those from all of the nanoparticle formulations (81.4±1.5% for 2-layer^1^-50, 94.4±1.2% for 2-layer^2^-50, 99.9±0.1% for 1-layer-50, 69.5±2.2% for 2-layer^1^-75, 84.7±4.0% for 2-layer^2^-75, and 99.1±1.0% for 1-layer-75). The increase in dissolution rate of progesterone in the latter is attributed to nanosizing which improved progesterone molecular dispersity, as demonstrated in the literature.^9^

### Analytical Model of Diffusive Release of Progesterone from Nanoparticles

To explain the release curves for progesterone from monolayer and core shell nanoparticles, we developed an analytical model incorporating a treatment of the low solubility of the drug, using the same methods as described in an earlier study.^43^ We consider particles in which an active ingredient is initially uniformly distributed, and diffuses with diffusion coefficient *D* out of a sphere of radius *a*. For the case of release into a finite volume we may assume that the concentration inside the particle, at its surface, is controlled by the concentration in the fluid and a partition coefficient *K*. The relative concentrations also involve the total volume of the particles *V*_p_ and the volume of the surrounding fluid *V*_f_. Thus, the equations to be solved are

(4) $${{\partial \left({{\rm{r}}{{\rm{c}}_{\rm{p}}}\left({{\rm{r}},{\rm{t}}} \right)} \right)} \over {\partial {\rm{t}}}} = {\rm{D}}{{{\partial ^2}\left({{\rm{r}}{{\rm{c}}_{\rm{p}}}\left({{\rm{r}},{\rm{t}}} \right)} \right)} \over {\partial {{\rm{r}}^2}}}$$

(5) $${{\rm{c}}_{\rm{p}}}\left({{\rm{r}},0} \right) = {{\rm{c}}_{{\rm{p}}0}}\,\,\,\,\,\,\,\,\,\,\,\,{\rm{ r}} \lt {\rm{b}}$$

(6) $${{\rm{c}}_{\rm{p}}}\left({{\rm{r}},0} \right) = 0\,\,\,\,\,\,\,\,\,\,\,\,\,\,\,\,\,\,\,\,\,{\rm{ b}} \le {\rm{r}} \lt {\rm{a}}$$

(7) $${{\rm{c}}_{\rm{p}}}\left({{\rm{a}},{\rm{t}}} \right) = {{\rm{K}}_{\rm{p}}}{{\rm{c}}_{\rm{f}}}\left({\rm{t}} \right)\,\,\,\,\,\,\,\,\,{\rm{ t}} \gt 0$$

(8) $${{\rm{c}}_{\rm{f}}}\left(0 \right) = 0$$

(9) $$ - {3 \over {\rm{a}}}{{\rm{V}}_{\rm{p}}}{\rm{D}}{\left. {{{\partial {{\rm{c}}_{\rm{p}}}\left({{\rm{r}},{\rm{t}}} \right))} \over {\partial {\rm{r}}}}} \right|_{{\rm{r}} = {\rm{a}}}} = {{\rm{V}}_{\rm{f}}}{{{\rm{d}}{{\rm{c}}_{\rm{f}}}\left({\rm{t}} \right)} \over {{\rm{dt}}}}\,\,\,\,\,\,\,\,\,\,\,\,\,\,\,{\rm{ t}} \gt 0$$

If we assume spherical symmetry, for a core shell particle with a core of radius a, the outer radius of particle is b. For the monolayer sphere, ${\rm{b}} = {\rm{a}}$, whereas for the core-shell structure we assume, because of the equal flow rates to the concentric feed tubes, a^3^ = b^3^ - a^3^, hence, we take b =0.8a so the volume of the core is equal to the volume of the shell. If the surrounding fluid is continuously sampled and refreshed, so that in a time interval of dt, a fraction fdt is replaced, the surface boundary condition becomes

(10) $$ - {3 \over {\rm{a}}}{{\rm{V}}_{\rm{p}}}{\rm{D}}{\left. {{{\partial {{\rm{c}}_{\rm{p}}}\left({{\rm{r}},{\rm{t}}} \right))} \over {\partial {\rm{r}}}}} \right|_{{\rm{r}} = {\rm{a}}}} = {{\rm{V}}_{\rm{f}}}{{{\rm{d}}{{\rm{c}}_{\rm{f}}}\left({\rm{t}} \right)} \over {{\rm{dt}}}} + {\rm{f'}}{{\rm{c}}_{\rm{f}}}\left({\rm{t}} \right)\,\,\,\,\,\,\,\,\,\,\,\,{\rm{ t}} \gt 0$$

Our previous work showed that *K, V*_p_ and *V*_f_ only entered the solution in the combination *K V*_p_/*V*_f_.^30^ If the sampling is discrete, a numerical solution is required, and we adopt a forward Euler method, in which we define the concentrations in the particle on a regularly spaced radial grid and take uniform time-steps. We use the Mathematica^®^ language, using 20 radial intervals with a time-step chosen to ensure stability of the numerical integration.

The radius of the particles is known, as are the sampling intervals and the fraction of fluid sampled and refreshed in the release measurements. Thus, the only unknowns are the diffusion coefficient *D* and the partition parameter K_ω_ = *K V*_p_/*V*_f_. We have made several calculations and found the optimal set of parameters for describing the drug release shown in Table 3.

**Table 3:** Optimal Physical Parameters for the Fractional Release Model for the Various Particle Configurations

Formulations	D (m^2^ s^−1^)	K_ω_
2-layer^1^-50	1.2×10^−17^	0.40
2-layer^2^-50	5.1×10^−18^	0.16
1-layer-50	4.2×10^−18^	0.063
2-layer^1^-75	1.2×10^−17^	0.63
2-layer^2^-75	4.0×10^−18^	0.25
1-layer-75	6.0×10^−18^	0.10

**Figure 8:**
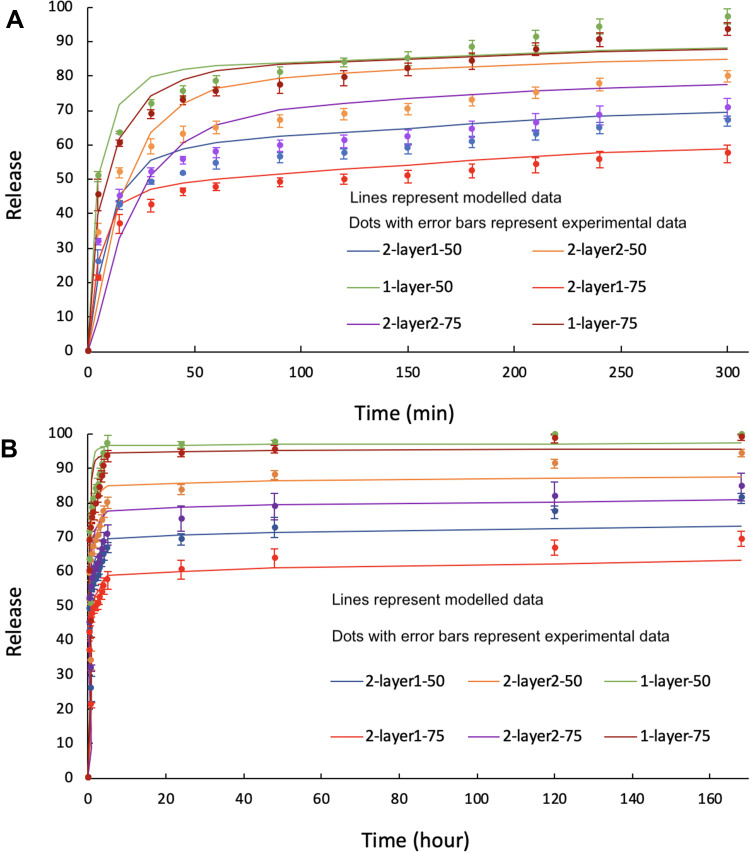
A comparison of the model (lines) and experimental (dots with error bars of the same color as the model lines): (**A**) early-stage, (**B**) Seven-day release from the 2-layer and 1-layer structures.

We note that the diffusion coefficients for progesterone in PLGA are similar to previously reported values which were between 1.0×10^−18^ m^2^s^−1^ and 1.4×10^−17^ m^2^s−1.^30^ With the values from Table 3 we obtain the release profiles shown in Figure 8A, which deals with the first five hours of early-stage release, and Figure 8B shows the entire tested seven-day release period. While the long-term predicted release profiles show good agreement with the experimental data, the details of the short-term release are not very accurately predicted, with the model (lines) tending to lie above the experimental results (points with error bars). We note that the partition factor K_ω_ of the monolayer particles is different from that for the core shell particles. This difference is attributed to the fact that the monolayer particles have a surface concentration of progesterone which allows for a significant burst release in the early stages whereas the core-shell structure places an additional diffusion barrier between the drug-loaded core material and the surrounding fluid which suppresses the initial burst release. Furthermore, the calculated results for the two-layered particles at short-term release are significantly better than those for the one-layered particles: this could be because of inhomogeneity in the distribution of the progesterone in the PLGA, which would have more of an effect at short times in the one-layer particles where release from near the surface can start as soon as the particles are immersed in fluid. The difference between single needle and coaxial needles might have an effect on the nanostructure of the particles, and this may be why the K_ω_ values are rather small for the one-layer particles. Work is in progress to explore the effect of core shell and three-layer nanoparticle configurations on a range of drug release behavior and further develop the analytical model to predict complex effects. For example, we note that drug diffusion during storage within the nanoparticle, pH values of the surrounding physiological environment, and enzymatic and bile salt effects are important aspects that can complicate the PLGA polymer behavior as well as the progesterone drug release behavior in a practical application. A recent study showed that PLGA can behave as a surfactant-dependent substrate for pancreatic lipase.^44^ Developing a deeper understanding of these biopharmaceutical aspects in the current formulations is an important future direction of this work. Furthermore, super-resolution confocal microscopy revealed a fuzzy boundary between the core and shell layers— whether this is due to imaging resolution limit or drug diffusion within the layered nano-structure requires careful further investigation. Work is in progress to assess the nanoparticle stability, and the manufacturing and storage conditions under which drug diffusion within the nano-structure may occur and to what extent, to develop manufacturing and storage protocols to minimize drug diffusion and maximize stability in the proposed nano-formulation. The nanoparticles once produced were collected and used as dry powders which could be placed in a capsule to be given as capsule dosage forms. The practical format to supply the nano-formulation to patients needs to be considered within the framework of commercialization and packaging. Future work will also compare with various other formulation methods such as oral lipid-based drug delivery systems and soft gel capsules to further understand the wider impact of the proposed formulation.

## Conclusions

The present investigation demonstrates that drug release can be significantly controlled by varying the hydrophilicity of the polymeric drug carrier through different monomer ratios in copolymer composition as well as monolayer and core shell drug compositions. Six formulations of progesterone were prepared by coaxial electrospray using PLGA of copolymer ratio 50:50 and 75:25 as drug excipients and achieved comparable nanoparticle diameters ranging from 472.1±54.8 to 588.0±92.1 nm. In vitro drug release study and computational modelling revealed that all six nanoparticle formulations improved the release of poorly water-soluble progesterone in comparison with control. Moreover, the initial burst release of progesterone decreased with lower hydrophilic copolymer GA ratio. Furthermore, the different core shell configurations strongly influenced the bioavailability and release behavior of progesterone: core shell formulations with progesterone co-dissolved in PLGA core exhibited enhanced release over five hours at 79.9±1.4% and 70.7±3.5% for LA:GA 50:50 and 75:25 in comparison with pure progesterone without polymer matrix in the core at 67.0±1.7% and 57.5±2.8%, respectively.

By encapsulating hydrophobic progesterone into PLGA nanoparticle matrices, the dissolution rate of the drug improved significantly in comparison with pure progesterone control because of nano-sizing which improved drug molecular dispersity. This work shows coaxial electrospray as an effective formulation technology. The six formulations with varying release rates are useful for developing nanomedicine strategies to enable sustained drug release in novel oral formulations using biomaterials to enhance the release of poorly water-soluble drugs like progesterone. Future work will further develop the computational model to predict complex release profiles and explore the effect of core shell and three-layer nanoparticle configurations in comparison with other formulation methods on the release of a range of drugs. Work is in progress to study manufacturing and storage effects on the stability and integrity of the nano-formulation as well as developing a deeper understanding of the biopharmaceutical aspects such as pH, micelles and enzymatic effects in the proposed formulations.
